# Current knowledge of Krüppel-like factor 5 and vascular remodeling: providing insights for therapeutic strategies

**DOI:** 10.1093/jmcb/mjaa080

**Published:** 2021-01-25

**Authors:** Ziyan Xie, Junye Chen, Chenyu Wang, Jiahao Zhang, Yanxiang Wu, Xiaowei Yan

**Affiliations:** 1 Department of Cardiology, Peking Union Medical College Hospital, Peking Union Medical College & Chinese Academy of Medical Sciences, Beijing 100730, China; 2 Department of Vascular Surgery, Peking Union Medical College Hospital, Peking Union Medical College & Chinese Academy of Medical Sciences, Beijing 100730, China

**Keywords:** Krüppel-like factor 5 (KLF5), vascular remodeling, inflammation, angiogenesis, drug development, microRNA

## Abstract

Vascular remodeling is a pathological basis of various disorders. Therefore, it is necessary to understand the occurrence, prevention, and treatment of vascular remodeling. Krüppel-like factor 5 (KLF5) has been identified as a significant factor in cardiovascular diseases during the last two decades. This review provides a mechanism network of function and regulation of KLF5 in vascular remodeling based on newly published data and gives a summary of its potential therapeutic applications. KLF5 modulates numerous biological processes, which play essential parts in the development of vascular remodeling, such as cell proliferation, phenotype switch, extracellular matrix deposition, inflammation, and angiogenesis by altering downstream genes and signaling pathways. Considering its essential functions, KLF5 could be developed as a potent therapeutic target in vascular disorders.

## Introduction 

Cardiovascular disease is the leading factor of disability and death all over the world. The understanding of disease pathogenesis remains unclear. Cardiovascular diseases often accompany vascular structure changes. Accumulating evidence has indicated vascular remodeling, a pathological basis for cardiovascular disease as well as other lethal diseases. Vascular remodeling contributes to occlusion, narrowing, stiffening, distension, or even rupture of blood vessels, thus impeding the physiologic functions of vessels (contraction and oxygenation) and leading to severe defects. It is of great significance to understand the molecular mechanisms and develop therapeutic targets for the prevention and treatment of vascular remodeling.

Krüppel-like factor 5 (KLF5) is a member of a zinc-finger (ZF)-containing transcription factor family consisting of 20 members that play critical roles in diverse biological processes. Its name, Krüppel, means ‘cripple’ in German, which was derived from a mutation in *Drosophila*, leading to their body patterning and segmentation (Nüsslein-Volhard and Wieschaus, 1980). Several KLF members have been proved to play vital roles in the progression of cardiovascular diseases, such as KLF2 (Chandra et al., 2011), KLF4 (Shankman et al., 2015), KLF11 (Yin et al., 2013), and KLF15 (Lu et al., 2013). In vascular smooth muscle cells (VSMCs), KLF5 was initially isolated from rabbit aorta and has been identified downregulated as development but re-induced in the neointima after injury in the adult aorta (Watanabe et al., 1999). KLF5 is highly expressed in human skin, esophagus, colon, and small intestine, but scarcely in the heart, brain, or spleen (Fagerberg et al., 2014).

KLF protein structures are highly conserved in their three Cys2His2-type ZF domains at the carboxyl-terminal ends. Its ZF domains could specifically bind to the GC-rich sequences located in the promoter of target genes, promoting DNA binding and nuclear localization (Basu et al., 2007). The functional diversity of KLF proteins is mostly carried out by the N-terminal ends, which facilitate specific protein‒protein binding, as well as repressive and active transcriptional modulation, and provide sites for post-translational modifications (PTMs) (McConnell and Yang, 2010). KLF5 contains a proline-rich transactivation domain (TAD) besides the three ZF domains at the amino-terminal end, allowing KLF5 to induce or suppress target gene expression (Nagai et al., 2005). The TAD in humans was suggested at the region of amino acids 324‒338 (Kojima et al., 1997). KLF5 could directly interact with a range of co-activators, co-repressors, and PTM regulators, thus acting together to modulate target gene expression in various biological processes. The interaction of KLF5 and the cofactor retinoic acid receptor-α (RARα) activates the expression of platelet-derived growth factor subunit A (PDGF-A), leading to VSMC proliferation (Kada et al., 2008). PTMs alter the protein level and transactivating function of KLF5. KLF5 can be phosphorylated on the sites Ser 153 (S153) and S303 in humans and S406 in rats by protein kinase C (PKC), glycogen synthase kinase 3β kinase, and p38, respectively (Zhang and Teng, 2003; Liu et al., 2010; Zhao et al., 2010). Phosphorylation promotes the transactivating function of KLF5. Acetylation at Lys 369 (K369) is executed by p300, while histone deacetylase 1 (HDAC1) and SET deacetylate KLF5 (Miyamoto et al., 2003; Matsumura et al., 2005). SUMOylation on K162 and K209 by enzyme SUMO1 converts KLF5 from a transcription activator to an inhibitor (Du et al., 2008; Oishi et al., 2008). KLF5 can also be ubiquitinated by E3 ubiquitin ligases WWP1 and FBW7, which does not influence the transcription ability but regulates the protein level of KLF5 (Chen et al., 2005; Zhao et al., 2010).

Studies on KLF5 have been going on for nearly two decades. KLF5 exerts considerable effects on cardiovascular diseases, such as atherosclerosis (Wang et al., 2019b), cardiac hypertrophy (Shindo et al., 2002), hypertension (Yao et al., 2008), and restenosis (Hoshino et al., 2000). The vascular role of KLF5 is first uncovered when it was reported to bind to the promoter of the smooth muscle myosin heavy chain (MHC) gene, *MYH10* (Watanabe et al., 1999). This review will focus on the pathological actions and regulations of KLF5 in cellular processes of vascular remodeling and summarize current gaps to propose potential research thoughts targeting KLF5.

## Roles of KLF5 in vascular remodeling

**Figure 1:**
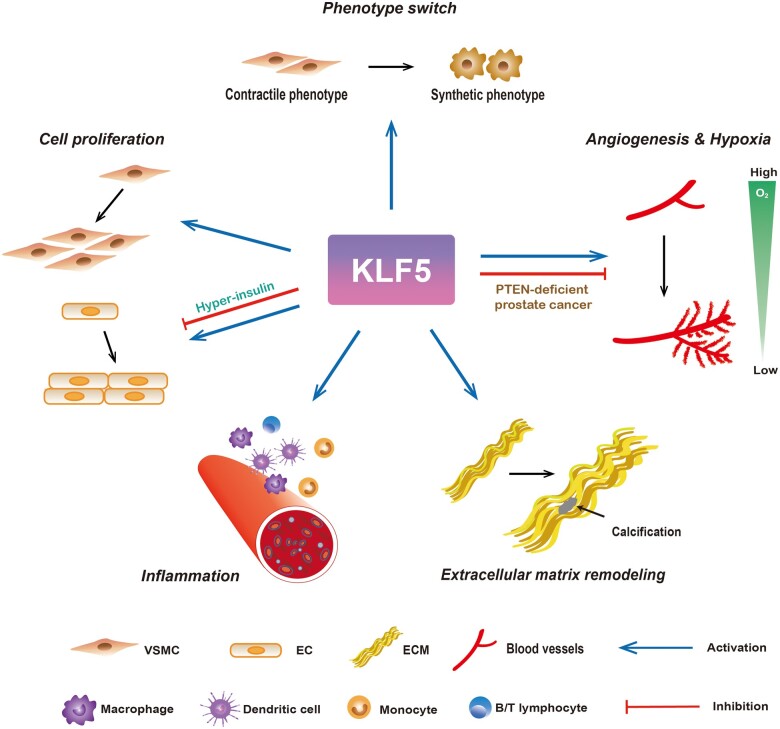
The roles of KLF5 in vascular remodeling. KLF5 induces the processes of cell phenotype switch, inflammation, and ECM remodeling, and exerts double-edged effects on cell proliferation and hypoxia-related angiogenesis, indicating a crucial role of KLF5 in vascular remodeling.

In native vessels, the vascular wall keeps in a quiescent, contractile state. Under pathologic conditions, vascular remodeling occurs, and the structure and function of the vessel wall are adjusted to accommodate the new environment, which is characterized by aberrant growth, thickening, and impaired elasticity of the layers of the vascular wall. In addition to cardiovascular diseases, vascular remodeling has also been observed in stroke, diabetes, and cancers (Fan et al., 2017). Vascular remodeling activates multiple intracellular signaling pathways to regulate vascular cell phenotype, proliferation, migration, apoptosis, extracellular matrix (ECM) synthesis and degradation, inflammation, and oxidative stress production. The expression of KLF5 protein was found re-induced in rat aortic neointimal smooth muscle cells to stimulate cell proliferation and angiogenesis after injury, revealing the promising role of KLF5 in vascular injury response (Watanabe et al., 1999). KLF5 heterozygous-deficient (*Klf5^+/−^*) mice showed attenuated neointimal formation, adventitia thickening, angiogenesis, and fibrosis (Shindo et al., 2002). Therefore, as a transcription factor, KLF5 regulates many genes involved in multiple biological processes contributing to vascular remodeling (Table 1) and exerts functions in smooth muscle cells as well as endothelial cells (ECs) and fibroblasts (Figure 1).

**Table 1:** Target genes of KLF5 in functional processes involved in vascular remodeling.

Function	Target genes	References
Proliferation	*CDKN1A*,* PDGFA*,* PDGFB*,* CCNA1*,* CCND1*,* CCNE1*,* PCNA*,* NOS3*	Nagai et al. (2005); He et al. (2009); Li et al. (2011); Gao et al. (2015a); Wang et al. (2019a, c)
Apoptosis	*BIRC5*,* CASP3*,* CASP9*	Bafford et al. (2006); Li et al. (2016a)
Phenotype switch	*CNN1*,* ACTA*,* TAGLN*,* MYH10*,* SERPINE1*,* EGR1*, *VEGFR*,* PDGFA*,* RUNX2*	Nagai et al. (2001); Fujiu et al. (2005); Zhang et al. (2014, 2016)
Inflammation	*IL1B*,* IL6*,* IL17*,* TNFA*,* CD38*,* CCL2*,* RELA*	Kumekawa et al. (2008); Jiang et al. (2015); Zhang et al. (2017a, 2018); Wang et al. (2019b)
Hypoxia	*HIF1A*	Li et al. (2016a)
Angiogenesis	*VEGFA*,* PDGFA*,* FGFBP1*,* TNFAIP2*,* AGGF1*,* NOS3*	Zheng et al. (2009); Gao et al. (2015b); Nakajima et al. (2016); Yang et al. (2017a); Jia et al. (2018); Wang et al. (2019c);
ECM remodeling	*MMP2*,* MMP3*,* MMP9*,* SERPINE1*,* RUNX2*	Delgado-Olguin et al. (2014); Zhang et al. (2014); Xu et al. (2018); Adnot et al. (2019)
Others	*ANO1*,* MYO9B*,* PPARG*,* PI3K*	Zhang et al. (2015); Li et al. (2016b); Ma et al. (2017b); Yang et al. (2017a)

### KLF5 and cell proliferation

Vascular cell proliferation is a crucial inducer of vascular remodeling. Vascular ECs locate in the inner layer of vessels, while VSMCs constitute the media, the major part of the vessel wall. The disrupted proliferation of any vascular cells may cause intima‒media thickening, undermining the homeostasis of the vessel wall. Numerous molecules participate in the regulation of vascular cell proliferation, which fall into three classes: growth factors like vascular endothelial growth factor (VEGF) and PDGF, cell cycle promoting proteins, such as cyclins (e.g. cyclin A, cyclin D, and cyclin E), cyclin-dependent kinases (CDKs), and cyclin-dependent kinase inhibitors (CKIs, e.g. p21 and p27), and DNA repair proteins such as proliferating cell nuclear antigen (PCNA).

As a transcription factor, KLF5 could alter the expression of genes involved in cell proliferation and apoptosis. Many studies demonstrated that KLF5 plays a driving role in the proliferation of VSMCs. Angiotensin II (Ang II) induces KLF5 expression through angiotensin II type 1 receptor (AT1R), PKC, mitogen-activated protein kinase (MAPK) pathway, and reactive oxygen species (ROS) activation in the VSMCs, which promotes cell proliferation in VSMCs (Gao et al., 2006). KLF5 suppresses p21 via interaction with c-Jun, thus inducing the proliferative response of Ang II (He et al., 2009). Additionally, KLF5 forms a complex with RARα and HDAC2 to block the expression of p21 (Zheng et al., 2011). The KLF5–p21 signaling is also activated in serine/arginine-rich splicing factor 1 (SRSF1)-mediated cell proliferation and neointima formation (Xie et al., 2017). Cooperating with p50, KLF5 activates the promoter of PDGF-A chain (Nagai et al., 2005). And KLF5 knockdown suppresses the expression of AT1R and PDGF-BB, thereby arresting cell cycle at the G0/G1 phase and inhibiting VSMC proliferation (Li et al., 2011). KLF5 activates cell cycle proteins as well, including cyclin A1, cyclin D1, and cyclin E1. Silencing of KLF5 leads to a remarkable reduction in cyclin D1 in VSMCs (Gao et al., 2015a). Transforming growth factor-β (TGF-β) inhibits KLF5-induced cyclin D1 expression via Smad, preventing the progression of VSMCs from G1 phase to S phase (Martin-Garrido et al., 2013). KLF5 is also a target gene of metastasis-associated lung adenocarcinoma transcript 1 (MALAT1); the MALAT1/hsa-miroRNA (miR)-124-3p.1/*KLF5* axis is pivotal in cell cycle progression of pulmonary artery hypertension. Suppression of any links in the axis results in decreased expression of PCNA, cyclin A1, cyclin D1, and cyclin E1 and arrests in cell cycle progression (Wang et al., 2019a). Moreover, the mutual interaction between KLF5 and survivin, an inhibitor of apoptosis protein, shows to promote cell proliferation and prevent apoptosis in veins and pulmonary arteries (Bafford et al., 2006; Hofmann et al., 2014). In rat basilar smooth muscle cells, TMEM16A, also a known downstream signal of KLF5, was reported to induce VSMC differentiation and inhibit VSMC proliferation by upregulating smooth muscle 22α (SM22α) and suppressing cyclin D (Wang et al., 2012). KLF5 inhibits TMEM16A transcription by blocking the positive feedback loop between myocardin and TMEM16A via competitive binding with myocardin, thus leading to VSMC proliferation (Zhang et al., 2015). KLF5 is also upregulated in intracranial aneurysms. Cell Counting Kit-8 assay indicates that KLF5 induces VSMC proliferation, resulting in aneurysms formation and growth (Xu et al., 2018). Beyond those roles, KLF5 is involved in VSMC contact inhibition. PI3-kinase/Akt/miR-145/KLF5 pathway contributes to the disruption of contact inhibition, which could be abrogated by KLF5 knockdown (Sun et al., 2018).

In ECs, knockdown of KLF5 downregulates PCNA, AT1R, and PDGF-BB, which dampens EC proliferation and results in attenuated intimal hyperplasia (Li et al., 2011). Additionally, KLF5 is shown to bind directly with the promoter of VEGF and induce transcription and protein formation. Decreased KLF5 attenuates VEGF mRNA expression and protein secretion (Zhou et al., 2019). However, KLF5 plays a different role in EC proliferation under hyperinsulinemia condition (Wang et al., 2019c). In type 2 diabetes mellitus mouse model, KLF5 is induced by high insulin level in the blood, and overexpression of KLF5 significantly inhibits nitric oxide synthase 3 (NOS3) transcription and diminishes the level of endothelial nitric oxide synthase (eNOS), thus compromising EC proliferation and blunting angiogenic response. Congruently, the previous study has reported that KLF5-overexpressing VSMCs transfer miR-155 by exosomes to ECs, causing inhibited proliferation/migration and disrupted tight junctions of ECs. The miR-155 transfer is triggered by KLF5 overexpression, which implies that KLF5 may be necessary in mediating the link between VSMCs and ECs (Zheng et al., 2017). Altogether, KLF5 stimulates VSMC proliferation and plays a double-edged role in the proliferation of ECs under certain circumstances, thus providing mechanistic evidence for intimal hyperplasia, media thickening, and other vascular pathological changes.

### KLF5 and phenotype switch

VSMCs usually stay in a quiescent and differentiated state, but they maintain a high degree of plasticity. Under pathological and experimental status, VSMCs migrate to the intima and shift from contractile phenotype to synthetic phenotype (Allahverdian et al., 2018). This phenomenon, named phenotype switch, is a major component of vascular neointimal formation. Synthetic VSMCs, also called dedifferentiated VSMCs, show a phenotype featured as loss of contractility, increased proliferation, reduced expression of contractile markers and secretion of elastase, as well as increased expression of metalloproteinase and proinflammatory factors. α-smooth muscle actin (α-SMA), SM22α, smooth muscle myosin heavy chains (SM-MHC), serum response factor (SRF), and calponin are typical contractile marker proteins reduced or lost during phenotype switch (Allahverdian et al., 2018). KLF5 is also reported to stimulate the switch of VSMCs to a synthetic phenotype. In animal models, VSMCs are highly differentiated in the injured arteries of *Klf5^+/−^* mice compared with control mice. The expression of KLF5 is also elevated in aortas of patients with thoracic aortic dissection and atherosclerosis (Yan et al., 2016; Zhang et al., 2016). Overexpression of KLF5 exhibits dedifferentiation of human aortic smooth muscle cells (HASMCs) characterized by downregulated contractile proteins and increased migration. Consistently, downregulating KLF5 abrogates the phenotype switch of HASMCs (Yan et al., 2016). KLF5 achieves phenotype switch by regulating the expression of smooth muscle marker genes (both contractile and synthetic) in direct and indirect ways. Knockdown of KLF5 suppresses the downregulation of SM-MHC and α-SMA in cultured VSMCs (Fujiu et al., 2005). Transient reporter transfection assays indicate that KLF5 increases the expression of *MYH10* gene by directly binding to its promoter. In addition to *MYH10*, KLF5 transactivates other genes predominant in synthetic SMCs including plasminogen activator inhibitor-1 (*PAI-1*), early growth response 1 (*EGR-1*), VEGF receptor (*VEGFR*), and *PDGF-A* (Nagai et al., 2001). Besides, KLF5 also directly binds to the promoter of runt-related transcription factor 2 (RUNX2) to activate its transcription, resulting in vascular calcification and the coupling of VSMC phenotypic switch (Zhang et al., 2014). Alternative splicing of KLF5 by DHX9-bridged YB-1 disrupts its nuclear localization and reduces its ability of transactivation, thus alleviating phenotype conversion from contractile to synthetic (Huan et al., 2019). Furthermore, the miR-145/KLF5/myocardin axis plays a crucial role in the phenotype switch (Zhang et al., 2016). Myocardin, a downstream signaling molecule of KLF5, facilitates contractile marker gene expression in an SRF-dependent manner (Huang et al., 2015). VSMCs from patients with atherosclerosis exhibit a significant upregulation of KLF5. The increase of KLF5 concomitantly suppresses the expression of the contractile markers α-SMA and calponin, along with downregulation of miR-145 and myocardin (Zhang et al., 2016). Silencing of KLF5 and upregulation of myocardin by miR-9 mimic hinder the progression of phenotype switch (Lu et al., 2019). In spontaneous hypertension, overexpression of complement 3 enhances the promoter activity of KLF5, inducing the switch to the synthetic phenotype of VSMCs (Negishi et al., 2018). Besides, KLF5 is critical in the tumor necrosis factor-α (TNF-α)-mediated phenotype switch. TNF-α strikingly induces the promoter activity of KLF5. Overexpression of KLF5 attenuates the expression of SM22α and α-SMA, and thus facilitates the loss of Ang II-induced contraction (Kim et al., 2015). The cross-regulation of KLF8 and KLF5 is also detected in the TNF-α-induced phenotypic switch of VSMCs. Suppressed KLF8 expression and increased KLF5 expression contribute to the reverse of contractile phenotype (Ha et al., 2017). In vein graft, VSMC phenotype switch is a significant component of pathological adaption. After implantation, a sudden increase of KLF5 and following GATA binding factor (GATA6) appears to reduce the transcription of contractile phenotype markers trangelin, α-actin, and MHC, resulting in the conversion of VSMC phenotype and remodeling response of vein graft (Klein et al., 2017). Phenotype switch is a critical factor of vascular remodeling, which influences the ability of cell proliferation and migration. Synthetic VSMCs could secrete ECM-related enzymes, cytokines, and cell adhesion molecules, thus boosting ECM degradation and immune cell recruitment and aggregation.

### KLF5 and inflammation

Inflammation includes three key parts: initiation of proinflammatory signals, macrophage infiltration, and leukocyte recruitment (Kreuger and Phillipson, 2016). Aberrant inflammation is a pivotal pathological basis of many vascular diseases. Under pathological stimuli, the immune reaction is activated, which leads to aggregation of immune cells in the target organs and secretion of cytokines, resulting in inflammatory responses and causing tissue injuries. In *Klf5^+/−^* mice, a reduced level of granulation tissue together with diminished inflammatory cells and microvessels was detected in cuff-injured arteries, compared with wild-type mice (Shindo et al., 2002). Increasing evidence demonstrates KLF5 as a critical mediator for proinflammatory response by regulating the production of various cytokines. Atherogenesis is termed as a process of chronic inflammation depending on complex interactions among VSMC proliferation, endothelial dysfunction, and lipid deposition (Boulanger et al., 2017). Upregulation of KLF5 significantly increases the expression of interleukin-1 (IL-1), IL-6, TNF-α, and CD38, accelerating the activation and infiltration of macrophages, leading to the pathogenesis of atherosclerosis (Wang et al., 2019b) and intracranial aneurysms (Zhang et al., 2018). KLF5 silence suppresses TNF-α-mediated monocyte chemoattractant protein-1 expression, which is a crucial cytokine contributing to the migration of monocytes into the intima, macrophage activation, and vascular inflammation (Kumekawa et al., 2008). Also, lipid deposition, plaque size, and intima/media ratio were significantly decreased in KLF5 knockout mice than those in wild-type mice (Zheng et al., 2018).

Furthermore, elevated expression of KLF5 is also found in portal hypertension, accompanied by increased IL-1β, IL-6, IL-17, and TNF-α. Inhibition of KLF5 prevents endothelial dysfunction and overexpression of proinflammatory cytokines, thus abrogating the progression of splenomegaly in portal hypertension (Jiang et al., 2015). Multiple studies imply the KLF5 induces proinflammatory response by activating NF-κB. KLF5 nitration by inducible NO synthase (iNOS)-mediated peroxynitrite production drives diabetic vascular inflammation through interaction with p50 subunit of NF-κB and cooperatively inducing the generation of TNF-α and IL-1β (Zhang et al., 2017a). Consistently, miR-145 inhibits LPS-induced NF-κB activation by targeting KLF5, and the overexpression of miR-145 suppresses macrophage infiltration in the diabetic mouse model (He et al., 2020). Vitamin D receptor competes with KLF5 for binding to NF-κB p50, thus alleviating LPS-induced macrophage proliferation (Ma et al., 2017a). Besides, KLF5-mediated Myo9b and downstream RhoA activation is involved in podosome formation and promotes macrophage migration during the development of abdominal aortic aneurysm (Ma et al., 2017b). In KLF5 knockdown mouse models, M2-type rather than M1-type macrophages are predominant in abdominal aortic aneurysm tissues, which exert an anti-inflammatory effect (Ma et al., 2017b). However, KLF5 plays a different role in cardiomyocyte inflammation. Overexpression of KLF5 ameliorates oxygen‒glucose deprivation- or reperfusion-induced myocardial inflammation through downregulating the levels of inflammatory cytokines (IL-1β, IL-6, IL-8, and TNF-α) and activating myocardial ischemia/reperfusion-protective proteins, peroxisome proliferator-activated receptor γ (PPARγ) and PPARγ-coactivator-1α (Li et al., 2016b). The dual roles of KLF5 in inflammation are dependent on tissues and stimuli and require further study.

### KLF5 and ECM remodeling

ECM remodeling is a process composed of degradation and re-synthesis. At first, ECM degradation executed mainly by matrix metalloproteinases (MMPs) leads to expansion and increases vascular wall compliance to accommodate the pressure load. As remodeling proceeds, ECM synthesis and reorganization lead to imbalance among structural elements in the vascular wall, such as elastin and collagen, thus contributing to vessel wall thickening, fibrosis, calcification, and finally stiffness (Lemarié et al., 2010). ECM degradation, which increases wall compliance but eventually results in rigidity and outward expansion, characterizes aortic aneurysm; re-organized ECM leading to vascular fibrosis, calcification, and stiffness is observed in hypertension and atherosclerosis. Therefore, ECM remodeling is recognized as a crucial process in the pathogenesis of multiple vascular diseases (Ma et al., 2020).

KLF5 is proposed to induce ECM degradation by promoting the expression and secretion of MMPs. Upregulation of KLF5 reverses the function of miR-143/145 in VSMCs, thus promoting the expression of MMP2 and MMP3, as well as the growth and development of intracranial aneurysm (Xu et al., 2018). Histone methyltransferase Ezh2 protects vasculature from ECM degradation by inhibiting the activity of MMP9 via epigenetic repression of KLF5 in the endothelium (Delgado-Olguin et al., 2014). The activating effect of KLF5 on NF-κB has been reported before (Ma et al., 2017a; Zhang et al., 2017a; He et al., 2020). The NF-κB pathway exerts essential effects on early ECM degradation. In TGF-α-deficient mice, reduced NF-κB activation causes repressed MMP activity and collagen deposition in aortic wall compared with wild-type mice (Lemarié et al., 2010). TMEM16A could inhibit ECM degradation via suppressing the expression of MMP9 by with-no-lysine 1 (WNK1) in VSMCs, thus preventing cerebrovascular remodeling during hypertension (Zeng et al., 2019). Consequently, KLF5 may also promote MMP activity and ECM degradation indirectly by targeting NF-κB and TMEM16A. Moreover, KLF5 silencing prevents the differentiation of fibroblasts into myofibroblasts and abrogates the release of MMPs and collagen from fibroblasts in pulmonary vessels of patients with chronic obstructive pulmonary disease (Abe et al., 2016).

On the other hand, KLF5 induces ECM reorganization and production. KLF5 activates the promoter of PAI-1, an inhibitor of fibrin degradation, thus contributing to ECM accumulation and leading to vascular wall thickening and cardiac hypertrophy (Nagai et al., 2003; Adnot et al., 2019). TGF-β, which lies downstream of KLF5, plays a crucial role in ECM deposition and fibrosis by regulating the synthesis of various ECM components like collagen, elastin, and fibronectin (Shindo et al., 2002; Meng et al., 2016). Besides, KLF5 is a key mediator of VSMC calcification. Overexpression of KLF5 greatly induces the transcription of *RUNX2* in VSMCs, a marker gene of bone morphogenic protein, which promotes the conversion of VSMCs into osteogenic cells and results in vascular calcification (Zhang et al., 2014). Furthermore, inflammation is also implied as a driver of ECM remodeling by breaking up the balance between proteolytic enzymes (MMPs) and their inhibitors TIMPs (Jain et al., 2014). KLF5 may be a core link between inflammation and ECM deposition. Overexpressed KLF5 dramatically increases the expression of inflammatory cytokines like IL-1, IL-6, and TNF-α. These cytokines trigger endothelial-to-mesenchymal transition (EndoMT), a process that ECs converse to myofibroblast-like cells, which then increases the expression of collagen and vimentin and secretion of MMPs, contributing to collagen deposition and crosslink (Stenmark et al., 2016). As a consequence, inflammation and ECM remodeling are not independent factors but have an association, and their cumulative effects eventually result in vascular remodeling and diseases.

### KLF5 and hypoxia

Hypoxia plays a critical role in triggering cardiovascular diseases. Several studies depict the master role of hypoxia-inducible factor-1 (HIF-1) in hypoxia-induced vascular remodeling (Patten et al., 2010). HIF-1 includes two structurally related subunits (HIF-1α and HIF-1β) and binds to the target gene promoters containing hypoxia response elements (HREs) (Chung et al., 2010). HIF-1α activation triggers vascular inflammation by recruiting macrophages in Ang II-induced vascular remodeling and promotes the progression of aortic dissection via induction of ECM degradation and elastic plate breakage (Lian et al., 2019).

KLF5‒HIF-1α interaction plays a vital role in the progression of hypoxia-induced pulmonary hypertension and pulmonary artery remodeling (Li et al., 2016a). The expression of KLF5 is upregulated by hypoxia in the pulmonary artery *in vivo* and *in vitro*. KLF5 acts as an upstream regulator of HIF-1α and increases its expression. The KLF5‒HIF-1α axis promotes cell proliferation and migration via activation of cyclin B1, cyclin D1, and survivin as well as suppression of caspase-3 and caspase-9. Interactions between KLF5 and HIF-1α have also been widely reported in cancers that KLF5 is upregulated and induces cell proliferation and angiogenesis.

### KLF5 and angiogenesis

Angiogenesis denotes the growth of new capillaries from existing ones, depending on the balance between pro-angiogenic and anti-angiogenic signals. It is a substantially ordered process based on the interactions between cytokines, growth factors, various kinds of cells (ECs, stromal cells, pericytes, etc.), ECM, and basement membrane (BM) (Chung et al., 2010). Aberrant angiogenesis due to excessive angiogenic signals forms tortuous and disorganized capillaries and facilitates the generation of tumor growth, vascular remodeling, and several disorders (Jain, 2003).

KLF5 heterozygous knockout and KLF5-siRNA-silencing mice not only reduce the expression of KLF5, but also exhibit low numbers of CD31-positive cells, which are the prognostic angiogenic marker representing the level of angiogenesis (Shindo et al., 2002). KLF5 regulates angiogenesis by modulating VEGF-A, an important pro-angiogenic factor. In bladder carcinoma, KLF5 promotes angiogenesis via binding to GC-boxes and CACCC elements of the VEGF-A promoter and increasing its expression (Gao et al., 2015b). *In vivo* experiment showed that inoculation of eukaryotic elongation factor 2-knockdown liver cancer cells into mouse front legs suppresses EC proliferation and tube formation via reducing the protein level of KLF5 and preventing KLF5 binding to the VEGF promoter (Zhou et al., 2019). Besides, *PDGF* and fibroblast growth factor (*FGF*), which are crucial in angiogenic signals mediating vessel sprouting initiation, stalk elongation, recruitment, and stabilization of mural cells and VSMCs, are both downstream target genes of KLF5 (Carmeliet and Jain, 2011). The reduction of KLF5 expression leads to decreased mRNA level of PDGF-A together with diminished CD31-positive region ratio, hence inhibiting angiogenesis and tumor growth of prostate cancer (Nakajima et al., 2016). KLF5 induces fibroblast growth factor binding protein (FGF-BP) expression by binding to a GC-box of the FGF-BP promoter. Increased FGF-BP promotes breast cell proliferation and angiogenesis in breast cancer (Zheng et al., 2009). This KLF5‒FGF-BP interaction is also involved in Yes-associated protein (YAP)-mediated tumorigenesis and angiogenesis in breast cancer. YAP overexpression increases KLF5 and its target gene expression levels, leading to new vessel formation, cell survival, and migration (Zhi et al., 2012). Besides, KLF5 promotes breast cancer angiogenesis in part through TNF-α-induced protein 2 (TNFAIP2), an angiogenetic factor increasing capillary tube formation, and KLF5 positively regulates TNFAIP2 by directly binding to its promoter (Jia et al., 2018). In addition, KLF5 boosts glioblastoma angiogenesis via enhancing the promoter activity of angiogenic factor with G-patch and FHA domain 1 (AGGF1), which is identified as a pro-angiogenic factor and associated with angiogenesis in various cancers. Knockdown of KLF5 also represses PI3K, AKT, and ERK1/2 activities, together with AGGF1, contributing to suppressed angiogenesis in glioblastoma (Yang et al., 2017a).

Conversely, in type 2 diabetes mellitus model, a hyper-insulin environment, both *in vivo* and *in vitro* experiments indicate that KLF5 attenuates VEGF-induced endothelial migration and proliferation and compromises angiogenic function by inhibiting the expression of NOS3 (Wang et al., 2019c). Moreover, PI3K/AKT pathway and expression of HIF-1α, VEGF, PDGF-A, and PDGF-B are activated by KLF5 loss and blocked by KLF5 overexpression in PTEN-deficient prostate cancer (Ci et al., 2015). KLF5 plays an anti-angiogenetic role, which is opposite to previous studies. Therefore, KLF5 may have a cell-specific bidirectional function.

Furthermore, inflammation and ECM degradation also participate in the angiogenetic process. Inflammatory cytokines initiate pericytes detachment and vessel branching (Whiteford et al., 2016); ECM provides newly formed sprouts with matrix support, and MMPs and proteases degrade the basement membrane and liberate angiogenetic factors like VEGF (Viallard and Larrivee, 2017). Hence, KLF5 exerts dual effects in angiogenesis by directly mediating the expression of angiogenetic factors or through indirect links like triggering inflammation and ECM remodeling.

## Modulations of KLF5 in vascular remodeling

**Figure 2:**
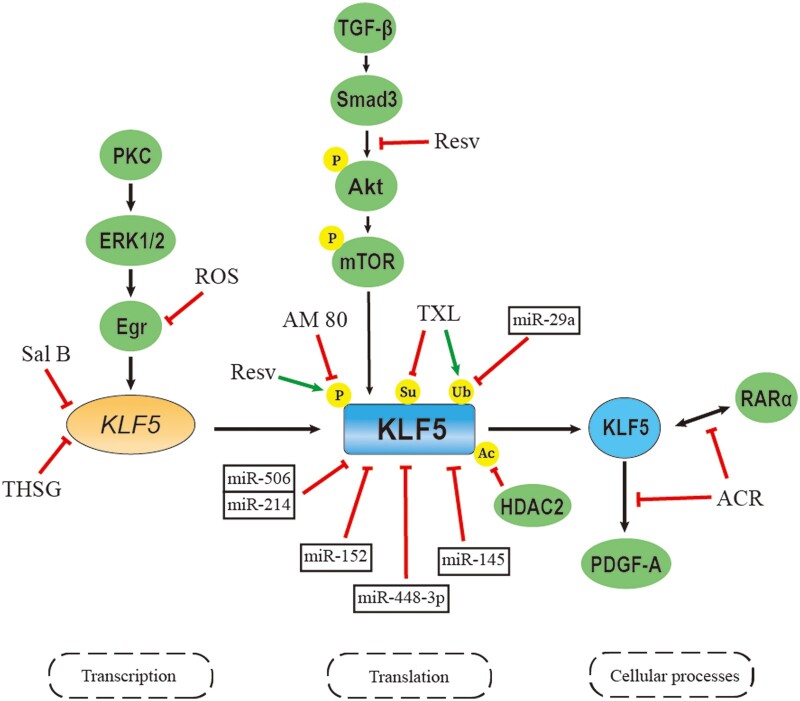
Agents targeting KLF5 for vascular disorder treatment. The expression and function of KLF5 are altered at the transcriptional, translational, and post-translational levels. Sal B, salvianolic acid B; Ros, rosiglitazone; Resv, resveratrol; TXL, tongxinluo; ACR, acyclic retinoid; P, phosphorylation; Ac, acetylation; Ub, ubiquitination; Su, SUMOylating.

KLF5 controls vascular homeostasis by regulating multiple cellular processes as discussed before. Therefore, modulators targeting KLF5 could be considered in pharmacologic application to alleviate vascular remodeling, thus decelerating or even halting the progression of vascular diseases. Here, we give a summary of modulators that regulate the expression and function of KLF5 during the process of vascular remodeling (Figure 2).

### Drugs

Several clinical trials targeting KLF5 activity have been reported to ameliorate vascular injury and remodeling. Salvianolic acid B is a widely used cardiovascular protective drug. It has been demonstrated to reverse neointimal hyperplasia and inhibit VSMC proliferation via decreasing the expression of KLF5, thus downregulating cyclin D1 (Zhao et al., 2019). In addition to cell proliferation, inflammation is also a crucial factor in neointimal hyperplasia. Tongxinluo, a traditional Chinese drug, prevents neointimal hyperplasia by inhibiting vascular inflammation. Tongxinluo exerts its anti-inflammatory effect via blocking KLF5 expression in macrophages and regulating KLF5 PTMs (ubiquitination and SUMOylating) (Jiang et al., 2016). Rosiglitazone is a kind of anti-diabetic medicine, which activates PPARγ. Rosiglitazone not only improves insulin resistance but also attenuates VSMC proliferation by interfering with the PKC/ERK1/2/Egr/KLF5 pathway. Egr could bind to the promoter of KLF5, and inhibition of Egr will then decrease the expression of KLF5 and following cyclin D1 (Gao et al., 2015a). Acyclic retinoid is first used as an anti-tumor agent in hepatoma. But it also modulates cardiovascular remodeling by abrogating interaction of KLF5 and RARα and repressing the transactivating function of KLF5 on PDGF-A chain promoter activity, subsequently inhibiting EC growth and angiogenesis (Kada et al., 2008). Another synthetic retinoic acid, AM80, has also been shown to inhibit the interaction of KLF5 and RARα in VSMCs by abrogating p38 signaling and subsequent KLF5 phosphorylation, which could be involved in therapeutic option for vascular remodeling (Fujiu et al., 2005). Resveratrol, a natural polyphenol from grapes, has been reported to hinder vascular cell proliferation and migration and inhibit VSMC phenotype switch. Resveratrol inhibits VSMC dedifferentiation by inhibiting mTOR pathway and blocking KLF5 protein production, which is considered as a novel anti-restenosis method (Zhu et al., 2017). Besides, resveratrol also participates in lipid metabolism by suppressing the expression of caveolin-1 (Cav-1), a kind of membrane protein major in regulating the transportation of cellular cholesterol and maintaining energy metabolism. Resveratrol activates KLF5 phosphorylation and inhibits the interaction of KLF5 and c-Myc, thus blocking the transactivation of Cav-1 by c-Myc and reversing the rising total cholesterol, triglyceride, and low-density lipoprotein cholesterol levels in high-fat diet-fed rats (Yang et al., 2017b). This function has not been tested in the context of cardiovascular diseases but provides new insight into the treatment of atherosclerosis and hyperlipidemia. Furthermore, a study indicates that 2,3,5,4-tetrahydroxystilbene-2-O-D-glucoside (THSG), a resveratrol analogue, has a vascular protective effect. It is also an upstream regulator of KLF5, which reduces KLF5 mRNA expression and blocks its downstream pathways (Duan et al., 2015).

### MicroRNAs

MicroRNAs (miRNAs) are a type of small (20‒25 nucleotides) non-coding RNAs regulating PTMs. Evidence from several studies has implied that miRNAs are essential factors in the mechanisms of vascular remodeling (Khachigian, 2019). miR-145 is a modulator of VSMC phenotype. It induces VSMC differentiation and protects against neointimal lesion formation via decreasing KLF5 expression and upregulating downstream myocardin (Cheng et al., 2009). Also, miR-145 promotes cardiac fibroblast transition to myofibroblasts in the same way by inhibiting the expression of KLF5 (Wang et al., 2014). miR-145-5p counteracts cell inflammation and apoptosis, at least in part via suppressing KLF5, thus blocking the formation and secretion of MMP2, MMP9, IL-6, IL-8, TNF-α, and CXCL8 (Dang et al., 2019). miR-152 decelerates the progression of atherosclerosis through inhibiting the secretion of inflammatory factors from macrophages by downregulating KLF5 (Wang et al., 2019b). miR-448-3p also regulates KLF5 directly in VSMCs. Downregulation of KLF5 by miR-448-3p represses vascular wall thickening and macrophage infiltration and activation, which alleviates the pathogenesis of intracranial aneurysms (Zhang et al., 2018). Besides, overexpression of miR-506 and miR-214 inhibits the expression of KLF4 and KLF5, thus protecting cardiomyocyte injury from ROS and cell apoptosis (Zhang et al., 2017b). Therefore, miRNAs may apply as biomarkers or therapeutic targets for vascular complications. Newly developed agents that alter miRNA activities, such as antisense oligonucleotides (Shen and Corey, 2018), miRNA mimics (Lu and Rothenberg, 2018), tiny LNA (seed-targeting 8-mer locked nucleic acid) (Obad et al., 2011), and miRNA sponges (Militello et al., 2017), are promising therapies but still need more clinical trials.

### Molecules targeting PTM of KLF5

PTMs modulate the protein level and activity of KLF5, which may inform the targeting of KLFs for therapy. Such modifications include phosphorylation, acetylation, ubiquitination, and SUMOylating (McConnell and Yang, 2010).

Phosphorylation of KLF5 could enhance its transactivating effect. Ang II induces KLF5 phosphorylation leading to an increased collaboration of KLF5 and c-Jun, subsequently inhibiting p21 expression and promoting VSMC proliferation (He et al., 2009). Dephosphorylation of KLF5 mediated by Am80 abrogates KLF5 binding to RARα that modulates various cellular processes (Zhang et al., 2009). The acetylation regulation of KLF5 was first demonstrated by Miyamoto et al. (2003) that p300 acetylated KLF5 whereas HDAC1 and SET deacetylated it. Succeeding findings reveal the effects of KLF5 acetylation on vascular remodeling. Acetylation of KLF5 augments the affinity of KLF5 and PARP-1, a pro-apoptotic enzyme, resulting in suppressed apoptosis of vascular cells (Suzuki et al., 2007). KLF5 deacetylation stimulated by HDAC2 prevents the binding between KLF5 and the p21 promoter, which compromises the suppressive effect of KLF5 on p21 expression (Zheng et al., 2011). Ras has also been documented as an upstream regulator of KLF5 acetylation that Ras alters interaction between KLF5 and p300 and suppresses KLF5 acetylation (Guo et al., 2019). Besides, ubiquitination usually downregulates KLF5 protein level and inhibits its activities. miR-29a improves KLF5 stability via decreasing the F-box and WD repeat domain containing 7 (Fbxw7)/CDC4-dependent ubiquitination of KLF5, which promotes VSMC proliferation and induces atherogenesis (Zheng et al., 2018). Moreover, drugs targeting PTMs of KLF5 have active functions in clinical treatment. Tongxinluo increases KLF5 ubiquitination by Fbxw7 and inhibits KLF5 SUMOylating by PI3K/Akt signaling, which facilitates the downregulation of KLF5 and relieves vascular inflammation (Jiang et al., 2016). Resveratrol ameliorates obesity and dyslipidemia via increasing KLF5 phosphorylation and interfering with the interaction between KLF5 and c-Myc, which subsequently modulates lipid metabolism balance (Yang et al., 2017b). Taken together, PTMs regulate KLF5 function and transcriptional activity, and a better understanding of PTMs is particularly important for the study of vascular disorders and the development of therapeutic agents.

## Limitations

Our review has demonstrated that KLF5 regulates various aspects of vascular remodeling. The crucial role of KLF5 in cardiovascular diseases has been first reported in 2002 (Shindo et al., 2002); since then, newly updated data have dug out more details of its role in diverse cellular processes and provided novel thinking for treatment application. However, the mechanisms of KLF5 action in vascular remodeling are still far from clear, and most approaches targeting KLF5 (e.g. miRNAs, antibodies, and inhibitors) in vascular disorder treatment are based on cell or animal experiments, while their clinical safety and effect are still obscure. The future challenges of miRNA therapy, including delivery safety, efficacy, site-directed targeting, and cellular stability, cannot be underestimated. Optimizing delivery system is also indispensable in treatments using chemically synthesized and natural products targeting KLF5. Besides, some drugs acting on KLF5 (e.g. rosiglitazone and resveratrol) are not specific, which target many other known function sites. Drugs like acyclic retinoid, which was demonstrated to alleviate the activity of KLF5, may become a potential therapy for cardiovascular diseases. However, clinical trials on acyclic retinoid in cardiovascular diseases are currently deficient. Furthermore, KLF5 plays a context-dependent role in different aspects. Opposing modulation of target genes accounts for the altered functions of KLF5. Accumulated evidence has shown that KLF5 can switch from a transactivator to transrepressor for the same sets of target genes (Guo et al., 2009; Parisi et al., 2010). A variety of studies have demonstrated that KLF5 promotes cell proliferation and angiogenesis via activating target genes, such as PDGF and VEGF. (Li et al., 2011; Zhou et al., 2019). Conversely, Ci et al. (2015) found that overexpression of KLF5 repressed the expression of HIF-1α, VEGF, PDGF-A, and PDGF-B, thus exerting an anti-angiogenetic and anti-proliferative effects in prostate cancer. This switch may be due to the PTM of KLF5 or the state and type of the cell. Additionally, hormones are also reported to be associated with the functions of KLF5. KLF5 dampens cell proliferation under estrogen treatment in estrogen receptor-positive breast cancer cells, while no significant effects have been shown in estrogen receptor-negative cancer cells and untreated cells (Guo et al., 2010). In a hyper-insulin environment, KLF5 plays an inhibitory role in EC proliferation by repressing the expression of VEGF and NOS3. It is postulated that high insulin level may alter KLF5 functions and adverse its effects on cell proliferation. Therefore, a deeper understanding of mechanistic knowledge and working on targeted therapy are of great importance.

## Perspectives

Vascular remodeling is strictly relevant to the progression of multiple vascular complications. Most vascular disorders, such as hypertension and atherosclerosis, have very complex etiologies. Therefore, studying the pathological mechanism of vascular disorders and exploring potential intervention targets are of great significance for the control and treatment of vascular diseases and reducing the damage to target organs.

KLF5 plays a promising role in the treatment of vascular disorders. Upstream regulators and molecules targeting PTMs of KLF5 in diverse signaling pathways could provide insight into therapeutic application. Co-immunoprecipitation, mass spectrometry, and RNA sequencing can be used to identify nuclear proteins and RNA directly interacted with KLF5 in cardiovascular diseases. Targeting protein–protein and protein‒RNA interactions is a solid way to develop effective inhibitors of transcription factors like KLF5. Besides, miRNAs have been documented as dramatic therapeutic targets. miRNAs regulate both the expression and activities of KLF5, and miRNA therapies have shown successful outcomes of cardiovascular disorders in preclinical experiments. It is reported that RNA modifications can influence the efficacy and specificity of miRNA via epi-transcriptomic changes. Therefore, further application of RNA modification on related miRNA of KLF5 may improve the feasibility of miRNA therapy in clinical practice. Given the cell-specific bidirectional property of KLF5, specific delivery is needed to ensure the safety and efficacy of therapeutic agents. For VSMCs, unique markers (e.g. α-SMA) and surface receptors could serve as delivery targets and improve the specificity. Moreover, epigenetic modifications are also critical in a deep understanding of mechanisms of vascular remodeling. Innovation of diagnostic, therapeutic, and preventive methods can be developed based on epigenetic modifications, such as gene silencing by DNA methylation. Potential methods or drugs could be developed to reverse the aberrant epigenetic modifications in vascular disorder treatment. We propose that with further studies, epigenetic changes of KLF5 expression can become biomarkers of vascular disorders, and targeted molecules (e.g. demethylating agents) will serve as therapeutic strategies. Additionally, to silence or regulate target gene expression (KLF5 or its upstream regulators), RNA interference-mediated methods (e.g. siRNA and shRNA) and gene editing technologies (e.g. CRISPR/Cas9) are favorable approaches, but more clinical trials are needed to prove their effects and safety in the human body.

KLF5 could also be developed as a biomarker for diagnosis in cardiovascular diseases. Research focusing on recognizing single-nucleotide polymorphisms (SNPs) or mutations is an attractive and novel way to find disease-associated genes and guide next-step treatment. Oishi et al. (2010) revealed that an SNP located at −1282 bp within the KLF5 functional locus is associated with human hypertension. Genetic variations contribute to the susceptibility of multiple diseases; genotyping and sequencing can help us uncover the mystery of genetic diseases and prevent them in advance.

As a result, identification of KLF5 binding partners and gene targets and elucidation of KLF5 biological functions will better develop prevention and therapy of vascular disorders.

## References

[mjaa080-B1] Abe K. , Sugiura H., Hashimoto Y., et al (2016). Possible role of Krüppel-like factor 5 in the remodeling of small airways and pulmonary vessels in chronic obstructive pulmonary disease. Respir. Res. 17, 7. 26792671 10.1186/s12931-016-0322-y PMC4719583

[mjaa080-B2] Adnot S. , Breau M., Houssaini A. (2019). PAI-1, a new target for controlling lung-cell senescence and fibrosis? Am. J. Respir. Cell Mol. Biol. *62*, 271–272. 10.1165/rcmb.2019-0341ED PMC7055697 31622556

[mjaa080-B3] Allahverdian S. , Chaabane C., Boukais K., et al (2018). Smooth muscle cell fate and plasticity in atherosclerosis. Cardiovasc. Res. 114, 540–550. 29385543 10.1093/cvr/cvy022 PMC5852505

[mjaa080-B4] Bafford R. , Sui X.X., Wang G., et al (2006). Angiotensin II and tumor necrosis factor-α upregulate survivin and Krüppel-like factor 5 in smooth muscle cells: potential relevance to vein graft hyperplasia. Surgery 140, 289–296. 16904982 10.1016/j.surg.2006.04.004

[mjaa080-B5] Basu P. , Lung T.K., Lemsaddek W., et al (2007). EKLF and KLF2 have compensatory roles in embryonic β-globin gene expression and primitive erythropoiesis. Blood 110, 3417–3425. 17675555 10.1182/blood-2006-11-057307 PMC2200909

[mjaa080-B6] Boulanger C.M. , Loyer X., Rautou P.E., et al (2017). Extracellular vesicles in coronary artery disease. Nat. Rev. Cardiol. 14, 259–272. 28150804 10.1038/nrcardio.2017.7

[mjaa080-B7] Carmeliet P. , Jain R.K. (2011). Molecular mechanisms and clinical applications of angiogenesis. Nature 473, 298–307. 21593862 10.1038/nature10144 PMC4049445

[mjaa080-B8] Chandra S.M. , Razavi H., Kim J., et al (2011). Disruption of the apelin-APJ system worsens hypoxia-induced pulmonary hypertension. Arterioscler. Thromb. Vasc. Biol. 31, 814–820. 21233449 10.1161/ATVBAHA.110.219980 PMC3113525

[mjaa080-B9] Chen C. , Sun X., Guo P., et al (2005). Human Krüppel-like factor 5 is a target of the E3 ubiquitin ligase WWP1 for proteolysis in epithelial cells. J. Biol. Chem. 280, 41553–41561. 16223724 10.1074/jbc.M506183200

[mjaa080-B10] Cheng Y. , Liu X., Yang J., et al (2009). MicroRNA-145, a novel smooth muscle cell phenotypic marker and modulator, controls vascular neointimal lesion formation. Circ. Res. 105, 158–166. 19542014 10.1161/CIRCRESAHA.109.197517 PMC2728297

[mjaa080-B11] Chung A.S. , Lee J., Ferrara N. (2010). Targeting the tumour vasculature: insights from physiological angiogenesis. Nat. Rev. Cancer 10, 505–514. 20574450 10.1038/nrc2868

[mjaa080-B12] Ci X. , Xing C., Zhang B., et al (2015). KLF5 inhibits angiogenesis in PTEN-deficient prostate cancer by attenuating AKT activation and subsequent HIF1α accumulation. Mol. Cancer 14, 91. 25896712 10.1186/s12943-015-0365-6 PMC4417294

[mjaa080-B13] Dang X. , Yang L., Guo J., et al (2019). miR-145-5p is associated with smoke-related chronic obstructive pulmonary disease via targeting KLF5. Chem. Biol. Interact. 300, 82–90. 30639269 10.1016/j.cbi.2019.01.011

[mjaa080-B14] Delgado-Olguin P. , Dang L.T., He D., et al (2014). Ezh2-mediated repression of a transcriptional pathway upstream of Mmp9 maintains integrity of the developing vasculature. Development 141, 4610–4617. 25359725 10.1242/dev.112607 PMC4302930

[mjaa080-B15] Du J.X. , Bialkowska A.B., McConnell B.B., et al (2008). SUMOylation regulates nuclear localization of Krüppel-like factor 5. J. Biol. Chem. 283, 31991–32002. 18782761 10.1074/jbc.M803612200 PMC2581587

[mjaa080-B16] Duan J. , Han X., Ling S., et al (2015). Aortic remodelling is improved by 2,3,5,4'-tetrahydroxystilbene-2-O-β-D-glucoside involving the Smad3 pathway in spontaneously hypertensive rats. Evid. Based Complement. Alternat. Med. 2015, 789027. 26693246 10.1155/2015/789027 PMC4677031

[mjaa080-B17] Fagerberg L. , Hallström B.M., Oksvold P., et al (2014). Analysis of the human tissue-specific expression by genome-wide integration of transcriptomics and antibody-based proteomics. Mol. Cell. Proteomics 13, 397–406. 10.1074/mcp.M113.035600 PMC3916642 24309898

[mjaa080-B18] Fan Y. , Lu H., Liang W., et al (2017). Krüppel-like factors and vascular wall homeostasis. J. Mol. Cell Biol. 9, 352–363. 28992202 10.1093/jmcb/mjx037 PMC5907833

[mjaa080-B19] Fujiu K. , Manabe I., Ishihara A., et al (2005). Synthetic retinoid Am80 suppresses smooth muscle phenotypic modulation and in-stent neointima formation by inhibiting KLF5. Circ. Res. 97, 1132–1141. 16224062 10.1161/01.RES.0000190613.22565.13

[mjaa080-B20] Gao D. , Hao G., Meng Z., et al (2015a). Rosiglitazone suppresses angiotensin II-induced production of KLF5 and cell proliferation in rat vascular smooth muscle cells. PLoS One 10, e0123724. 25874449 10.1371/journal.pone.0123724 PMC4397085

[mjaa080-B21] Gao D. , Niu X., Ning N., et al (2006). Regulation of angiotensin II-Induced Krüppel-like factor 5 expression in vascular smooth muscle cells. Biol. Pharm. Bull. 29, 2004–2008. 17015941 10.1248/bpb.29.2004

[mjaa080-B22] Gao Y. , Wu K., Chen Y., et al (2015b). Beyond proliferation: KLF5 promotes angiogenesis of bladder cancer through directly regulating VEGFA transcription. Oncotarget 41, 43791–43805. 10.18632/oncotarget.6101 PMC4791267 26544730

[mjaa080-B23] Guo P. , Dong X.-Y., Zhao K.-W., et al (2010). Estrogen-induced interaction between KLF5 and estrogen receptor (ER) suppresses the function of ER in ER-positive breast cancer cells. Int. J. Cancer 126, 81–89. 19569049 10.1002/ijc.24696 PMC2783791

[mjaa080-B24] Guo P. , Xing C., Fu X., et al (2019). Ras inhibits TGF-β-induced KLF5 acetylation and transcriptional complex assembly via regulating SMAD2/3 phosphorylation in epithelial cells. J. Cell. Biochem. *121*, 2197–2208. 31724223 10.1002/jcb.29443

[mjaa080-B25] Guo P. , Zhao K.-W., Dong X.-Y., et al (2009). Acetylation of KLF5 alters the assembly of p15 transcription factors in transforming growth factor-β-mediated induction in epithelial cells. J. Biol. Chem. 284, 18184–18193. 19419955 10.1074/jbc.M109.007096 PMC2709394

[mjaa080-B26] Ha J.M. , Yun S.J., Jin S.Y., et al (2017). Regulation of vascular smooth muscle phenotype by cross-regulation of krüppel-like factors. Korean J. Physiol. Pharmacol. 21, 37–44. 28066139 10.4196/kjpp.2017.21.1.37 PMC5214909

[mjaa080-B27] He M. , Han M., Zheng B., et al (2009). Angiotensin II stimulates KLF5 phosphorylation and its interaction with c-Jun leading to suppression of p21 expression in vascular smooth muscle cells. J. Biochem. 146, 683–691. 19628677 10.1093/jb/mvp115

[mjaa080-B28] He M. , Wu N., Leong M.C., et al (2020). miR-145 improves metabolic inflammatory disease through multiple pathways. J. Mol. Cell Biol. *12*, 152–162. 30941422 10.1093/jmcb/mjz015 PMC7109608

[mjaa080-B29] Hofmann A.D. , Takahashi T., Duess J.W., et al (2014). Increased pulmonary vascular expression of Krüppel-like factor 5 and activated survivin in experimental congenital diaphragmatic hernia. Pediatr. Surg. Int. 30, 1191–1197. 25323860 10.1007/s00383-014-3606-7

[mjaa080-B30] Hoshino Y. , Kurabayashi M., Kanda T., et al (2000). Regulated expression of the BTEB2 transcription factor in vascular smooth muscle cells: analysis of developmental and pathological expression profiles shows implications as a predictive factor for restenosis. Circulation 102, 2528–2534. 11076828 10.1161/01.cir.102.20.2528

[mjaa080-B31] Huan W. , Zhang J., Li Y., et al (2019). Involvement of DHX9/YB-1 complex induced alternative splicing of Krüppel-like factor 5 mRNA in phenotypic transformation of vascular smooth muscle cells. Am. J. Physiol. Cell Physiol. 317, C262–C269. 31116584 10.1152/ajpcell.00067.2019

[mjaa080-B32] Huang J. , Wang T., Wright A. C., et al (2015). Myocardin is required for maintenance of vascular and visceral smooth muscle homeostasis during postnatal development. Proc. Natl Acad. Sci. USA 112, 4447–4452. 25805819 10.1073/pnas.1420363112 PMC4394251

[mjaa080-B33] Jain R.K. (2003). Molecular regulation of vessel maturation. Nat. Med. 9, 685–693. 12778167 10.1038/nm0603-685

[mjaa080-B34] Jain S. , Khera R., Corrales-Medina V. F., et al (2014). Inflammation and arterial stiffness in humans. Atherosclerosis 237, 381–390. 25463062 10.1016/j.atherosclerosis.2014.09.011

[mjaa080-B35] Jia L. , Shi Y., Wen Y., et al (2018). The roles of TNFAIP2 in cancers and infectious diseases. J. Cell. Mol. Med. 22, 5188–5195. 30145807 10.1111/jcmm.13822 PMC6201362

[mjaa080-B36] Jiang B. , Deng Q., Huo Y., et al (2015). Endothelial Gab1 deficiency aggravates splenomegaly in portal hypertension independent of angiogenesis. Am. J. Physiol. Gastrointest. Liver Physiol. 308, G416–G426. 25501549 10.1152/ajpgi.00292.2014

[mjaa080-B37] Jiang W. , Zheng B., Zhang X.H., et al (2016). Tongxinluo inhibits neointimal formation by regulating the expression and post-translational modification of KLF5 in macrophages. Am. J. Transl. Res. 8, 4778–4790. 27904679 PMC5126321

[mjaa080-B38] Kada N. , Suzuki T., Aizawa K., et al (2008). Acyclic retinoid inhibits functional interaction of transcription factors Krüppel-like factor 5 and retinoic acid receptor-α. FEBS Lett*.* 582, 1755–1760. 18466769 10.1016/j.febslet.2008.04.040

[mjaa080-B39] Khachigian L.M. (2019). Transcription factors targeted by miRNAs regulating smooth muscle cell growth and intimal thickening after vascular injury. Int. J. Mol. Sci. 20, 5445. 10.3390/ijms20215445 PMC6861964 31683712

[mjaa080-B40] Kim S.H. , Yun S.J., Kim Y.H., et al (2015). Essential role of krüppel-like factor 5 during tumor necrosis factor α-induced phenotypic conversion of vascular smooth muscle cells. Biochem. Biophys. Res. Commun. 463, 1323–1327. 26102029 10.1016/j.bbrc.2015.06.123

[mjaa080-B41] Klein B. , Destephens A., Dumeny L., et al (2017). Hemodynamic influence on smooth muscle cell kinetics and phenotype during early vein graft adaptation. Ann. Biomed. Eng. 45, 644–655. 27624660 10.1007/s10439-016-1725-0 PMC5332303

[mjaa080-B42] Kojima S. , Kobayashi A., Gotoh O., et al (1997). Transcriptional activation domain of human BTEB2, a GC box-binding factor. J. Biochem. 121, 389–396. 9089417 10.1093/oxfordjournals.jbchem.a021600

[mjaa080-B43] Kreuger J. , Phillipson M. (2016). Targeting vascular and leukocyte communication in angiogenesis, inflammation and fibrosis. Nat. Rev. Drug Discov. 15, 125–142. 26612664 10.1038/nrd.2015.2

[mjaa080-B44] Kumekawa M. , Fukuda G., Shimizu S., et al (2008). Inhibition of monocyte chemoattractant protein-1 by Krüppel-like factor 5 small interfering RNA in the tumor necrosis factor α-activated human umbilical vein endothelial cells. Biol. Pharm. Bull. 31, 1609–1613. 18670098 10.1248/bpb.31.1609

[mjaa080-B45] Lemarié C.A. , Tharaux P.L., Lehoux S. (2010). Extracellular matrix alterations in hypertensive vascular remodeling. J. Mol. Cell. Cardiol. 48, 433–439. 19837080 10.1016/j.yjmcc.2009.09.018

[mjaa080-B46] Li D. , Ma S., Yang Y., et al (2011). BTEB2 knockdown suppresses neointimal hyperplasia in a rat artery balloon injury model. Mol. Med. Rep. 4, 413–417. 21468585 10.3892/mmr.2011.438

[mjaa080-B47] Li X. , He Y., Xu Y., et al (2016a). KLF5 mediates vascular remodeling via HIF-1α in hypoxic pulmonary hypertension. Am. J. Physiol. Lung Cell. Mol. Physiol. 310, L299–L310. 26702149 10.1152/ajplung.00189.2015

[mjaa080-B48] Li Y. , Li J., Hou Z., et al (2016b). KLF5 overexpression attenuates cardiomyocyte inflammation induced by oxygen-glucose deprivation/reperfusion through the PPARγ/PGC-1α/TNF-α signaling pathway. Biomed. Pharmacother. 84, 940–946. 27764756 10.1016/j.biopha.2016.09.100

[mjaa080-B49] Lian G. , Li X., Zhang L., et al (2019). Macrophage metabolic reprogramming aggravates aortic dissection through the HIF1α–ADAM17 pathway. EBioMedicine 49, 291–304. 31640947 10.1016/j.ebiom.2019.09.041 PMC6945268

[mjaa080-B50] Liu Y. , Wen J.-K., Dong L.-H., et al (2010). Krüppel-like factor (KLF) 5 mediates cyclin D1 expression and cell proliferation via interaction with c-Jun in Ang II-induced VSMCs. Acta Pharmacol. Sin. 31, 10–18. 20037604 10.1038/aps.2009.185 PMC4002698

[mjaa080-B51] Lu T.X. , Rothenberg M.E. (2018). MicroRNA. J. Allergy Clin. Immunol. 141, 1202–1207. 29074454 10.1016/j.jaci.2017.08.034 PMC5889965

[mjaa080-B52] Lu X. , Ma S. T., Zhou B., et al (2019). MiR-9 promotes the phenotypic switch of vascular smooth muscle cells by targeting KLF5. Turk. J. Med. Sci. 49, 928–938. 31122000 10.3906/sag-1710-173 PMC7018344

[mjaa080-B53] Lu Y. , Zhang L., Liao X., et al (2013). Krüppel-like factor 15 is critical for vascular inflammation. J. Clin. Invest*.* 123, 4232–4241. 23999430 10.1172/JCI68552 PMC3785338

[mjaa080-B54] Ma D. , Zhang R. N., Wen Y., et al (2017a). 1, 25(OH)_2_D_3_-induced interaction of vitamin D receptor with p50 subunit of NF-κB suppresses the interaction between KLF5 and p50, contributing to inhibition of LPS-induced macrophage proliferation. Biochem. Biophys. Res. Commun. 482, 366–374. 27856242 10.1016/j.bbrc.2016.11.069

[mjaa080-B55] Ma D. , Zheng B., Suzuki T., et al (2017b). Inhibition of KLF5‒Myo9b‒RhoA pathway-mediated podosome formation in macrophages ameliorates abdominal aortic aneurysm. Circ. Res. 120, 799–815. 28115390 10.1161/CIRCRESAHA.116.310367

[mjaa080-B56] Ma Z. , Mao C., Jia Y., et al (2020). Extracellular matrix dynamics in vascular remodeling. Am. J. Physiol. Cell Physiol. 319, C481–C499. 32579472 10.1152/ajpcell.00147.2020 PMC7509265

[mjaa080-B57] Martin-Garrido A. , Williams H. C., Lee M., et al (2013). Transforming growth factor β inhibits platelet derived growth factor-induced vascular smooth muscle cell proliferation via Akt-independent, Smad-mediated cyclin D1 downregulation. PLoS One 8, e79657. 24236150 10.1371/journal.pone.0079657 PMC3827379

[mjaa080-B58] Matsumura T. , Suzuki T., Aizawa K., et al (2005). The deacetylase HDAC1 negatively regulates the cardiovascular transcription factor Krüppel-like factor 5 through direct interaction. J. Biol. Chem. 280, 12123–12129. 15668237 10.1074/jbc.M410578200

[mjaa080-B59] McConnell B.B. , Yang V.W. (2010). Mammalian Krüppel-like factors in health and diseases. Physiol. Rev. 90, 1337–1381. 20959618 10.1152/physrev.00058.2009 PMC2975554

[mjaa080-B60] Meng X.-M. , Nikolic-Paterson D.J., Lan H.Y. (2016). TGF-β: the master regulator of fibrosis. Nat. Rev. Nephrol. 12, 325–338. 27108839 10.1038/nrneph.2016.48

[mjaa080-B61] Militello G. , Weirick T., John D., et al (2017). Screening and validation of lncRNAs and circRNAs as miRNA sponges. Brief. Bioinf*.* 18, 780–788. 10.1093/bib/bbw053 27373735

[mjaa080-B62] Miyamoto S. , Suzuki T., Muto S., et al (2003). Positive and negative regulation of the cardiovascular transcription factor KLF5 by p300 and the oncogenic regulator SET through interaction and acetylation on the DNA-binding domain. Mol. Cell. Biol. 23, 8528–8541. 14612398 10.1128/MCB.23.23.8528-8541.2003 PMC262669

[mjaa080-B63] Nagai R. , Shindo T., Manabe I., et al (2003). KLF5/BTEB2, a Krüppel-like zinc-finger type transcription factor, mediates both smooth muscle cell activation and cardiac hypertrophy. Adv. Exp. Med. Biol. *538*, 57–65. 10.1007/978-1-4419-9029-7_5 15098654

[mjaa080-B64] Nagai R. , Suzuki T., Aizawa K., et al (2001). Phenotypic modulation of vascular smooth muscle cells: dissection of transcriptional regulatory mechanisms. Ann. NY Acad. Sci. 947, 56–66. 11795310 10.1111/j.1749-6632.2001.tb03930.x

[mjaa080-B65] Nagai R. , Suzuki T., Aizawa K., et al (2005). Significance of the transcription factor KLF5 in cardiovascular remodeling. J. Thromb. Haemost. 3, 1569–1576. 16102021 10.1111/j.1538-7836.2005.01366.x

[mjaa080-B66] Nakajima Y. , Osakabe A., Waku T., et al (2016). Estrogen exhibits a biphasic effect on prostate tumor growth through the estrogen receptor β-KLF5 pathway. Mol. Cell. Biol. 36, 144–156. 26483416 10.1128/MCB.00625-15 PMC4702593

[mjaa080-B67] Negishi E. , Fukuda N., Otsuki T., et al (2018). Involvement of complement 3 in the salt-sensitive hypertension by activation of renal renin-angiotensin system in spontaneously hypertensive rats. Am. J. Physiol. Renal Physiol. 315, F1747–F1758. 30256128 10.1152/ajprenal.00370.2018

[mjaa080-B68] Nüsslein-Volhard C. , Wieschaus E. (1980). Mutations affecting segment number and polarity in Drosophila. Nature 287, 795–801. 6776413 10.1038/287795a0

[mjaa080-B69] Obad S. , dos Santos C.O., Petri A., et al (2011). Silencing of microRNA families by seed-targeting tiny LNAs. Nat. Genet. 43, 371–378. 21423181 10.1038/ng.786 PMC3541685

[mjaa080-B70] Oishi Y. , Manabe I., Imai Y., et al (2010). Regulatory polymorphism in transcription factor KLF5 at the MEF2 element alters the response to angiotensin II and is associated with human hypertension. FASEB J. 24, 1780–1788. 20086047 10.1096/fj.09-146589

[mjaa080-B71] Oishi Y. , Manabe I., Tobe K., et al (2008). SUMOylation of Krüppel-like transcription factor 5 acts as a molecular switch in transcriptional programs of lipid metabolism involving PPAR-δ. Nat. Med. 14, 656–666. 18500350 10.1038/nm1756

[mjaa080-B72] Parisi S. , Cozzuto L., Tarantino C., et al (2010). Direct targets of Klf5 transcription factor contribute to the maintenance of mouse embryonic stem cell undifferentiated state. BMC Biol. 8, 128. 20875108 10.1186/1741-7007-8-128 PMC2955566

[mjaa080-B73] Patten D.A. , Lafleur V.N., Robitaille G.A., et al (2010). Hypoxia-inducible factor-1 activation in nonhypoxic conditions: the essential role of mitochondrial-derived reactive oxygen species. Mol. Biol. Cell 21, 3247–3257. 20660157 10.1091/mbc.E10-01-0025 PMC2938389

[mjaa080-B74] Shankman L.S. , Gomez D., Cherepanova O.A., et al (2015). KLF4-dependent phenotypic modulation of smooth muscle cells has a key role in atherosclerotic plaque pathogenesis. Nat. Med. 21, 628–637. 25985364 10.1038/nm.3866 PMC4552085

[mjaa080-B75] Shen X. , Corey D.R. (2018). Chemistry, mechanism and clinical status of antisense oligonucleotides and duplex RNAs. Nucleic Acids Res. 46, 1584–1600. 29240946 10.1093/nar/gkx1239 PMC5829639

[mjaa080-B76] Shindo T. , Manabe I., Fukushima Y., et al (2002). Krüppel-like zinc-finger transcription factor KLF5/BTEB2 is a target for angiotensin II signaling and an essential regulator of cardiovascular remodeling. Nat. Med. 8, 856–863. 12101409 10.1038/nm738

[mjaa080-B77] Stenmark K.R. , Frid M., Perros F. (2016). Endothelial-to-mesenchymal transition: an evolving paradigm and a promising therapeutic target in PAH. Circulation 133, 1734–1737. 27045137 10.1161/CIRCULATIONAHA.116.022479 PMC4866805

[mjaa080-B78] Sun Y.Y. , Qin S.S., Cheng Y.H., et al (2018). MicroRNA expression profile and functional analysis reveal their roles in contact inhibition and its disruption switch of rat vascular smooth muscle cells. Acta Pharmacol. Sin. 39, 885–892. 29698390 10.1038/aps.2018.6 PMC5943918

[mjaa080-B79] Suzuki T. , Nishi T., Nagino T., et al (2007). Functional interaction between the transcription factor Krüppel-like factor 5 and poly(ADP-ribose) polymerase-1 in cardiovascular apoptosis. J. Biol. Chem. 282, 9895–9901. 17283079 10.1074/jbc.M608098200

[mjaa080-B80] Viallard C. , Larrivee B. (2017). Tumor angiogenesis and vascular normalization: alternative therapeutic targets. Angiogenesis 20, 409–426. 28660302 10.1007/s10456-017-9562-9

[mjaa080-B81] Wang D. , Xu H., Wu B., et al (2019a). Long non-coding RNA MALAT1 sponges miR-124-3p.1/KLF5 to promote pulmonary vascular remodeling and cell cycle progression of pulmonary artery hypertension. Int. J. Mol. Med**.** 44, 871–884. 31257528 10.3892/ijmm.2019.4256 PMC6657969

[mjaa080-B82] Wang M. , Yang H., Zheng L.Y., et al (2012). Downregulation of TMEM16A calcium-activated chloride channel contributes to cerebrovascular remodeling during hypertension by promoting basilar smooth muscle cell proliferation. Circulation 125, 697–707. 22215857 10.1161/CIRCULATIONAHA.111.041806

[mjaa080-B83] Wang W. , Zhang Y., Wang L., et al (2019b). mircroRNA-152 prevents the malignant progression of atherosclerosis via down-regulation of KLF5. Biomed. Pharmacother. 109, 2409–2414. 30551500 10.1016/j.biopha.2018.08.014

[mjaa080-B84] Wang X.H. , Yan C.Y., Liu J.R. (2019c). Hyperinsulinemia-induced KLF5 mediates endothelial angiogenic dysfunction in diabetic endothelial cells. J. Mol. Histol. 50, 239–251. 31049798 10.1007/s10735-019-09821-3

[mjaa080-B85] Wang Y.S. , Li S.H., Guo J., et al (2014). Role of miR-145 in cardiac myofibroblast differentiation. J. Mol. Cell. Cardiol. 66, 94–105. 24001939 10.1016/j.yjmcc.2013.08.007

[mjaa080-B86] Watanabe N. , Kurabayashi M., Shimomura Y., et al (1999). BTEB2, a Krüppel-like transcription factor, regulates expression of the SMemb_Nonmuscle myosin heavy chain B (SMemb_NMHC-B) gene. Circ. Res. 85, 182–191. 10417400 10.1161/01.res.85.2.182

[mjaa080-B87] Whiteford J.R. , De Rossi G., Woodfin A. (2016). Mutually supportive mechanisms of inflammation and vascular remodeling. Int. Rev. Cell Mol. Biol. 326, 201–278. 27572130 10.1016/bs.ircmb.2016.05.001

[mjaa080-B88] Xie N. , Chen M., Dai R., et al (2017). SRSF1 promotes vascular smooth muscle cell proliferation through a △133p53/EGR1/KLF5 pathway. Nat. Commun. 8, 16016. 28799539 10.1038/ncomms16016 PMC5561544

[mjaa080-B89] Xu J. , Yan S., Tan H., et al (2018). The miR-143/145 cluster reverses the regulation effect of KLF5 in smooth muscle cells with proliferation and contractility in intracranial aneurysm. Gene 679, 266–273. 30201338 10.1016/j.gene.2018.09.010

[mjaa080-B90] Yan Y. , Tan M.W., Xue X., et al (2016). Involvement of Oct4 in the pathogenesis of thoracic aortic dissection via inducing the dedifferentiated phenotype of human aortic smooth muscle cells by directly upregulating KLF5. J. Thorac. Cardiovasc. Surg. 152, 820–829.e4. 27353340 10.1016/j.jtcvs.2016.05.036

[mjaa080-B91] Yang C. , Zheng J., Xue Y., et al (2017a). The effect of MCM3AP-AS1/miR-211/KLF5/AGGF1 axis regulating glioblastoma angiogenesis. Front. Mol. Neurosci. 10, 437. 29375300 10.3389/fnmol.2017.00437 PMC5767169

[mjaa080-B92] Yang H. , Chen Q., Sun F., et al (2017b). Down-regulation of the klf5‒c-Myc interaction due to klf5 phosphorylation mediates resveratrol repressing the caveolin-1 transcription through the PI3K/PKD1/Akt pathway. PLoS One 12, e0189156. 29211809 10.1371/journal.pone.0189156 PMC5718516

[mjaa080-B93] Yao E.H. , Fukuda N., Ueno T., et al (2008). Complement 3 activates the KLF5 gene in rat vascular smooth muscle cells. Biochem. Biophys. Res. Commun. 367, 468–473. 18178156 10.1016/j.bbrc.2007.12.160

[mjaa080-B94] Yin K.-J. , Fan Y., Hamblin M., et al (2013). KLF11 mediates PPARγ cerebrovascular protection in ischaemic stroke. Brain 136, 1274–1287. 23408111 10.1093/brain/awt002 PMC3613710

[mjaa080-B95] Zeng X.L. , Sun L., Zheng H.Q., et al (2019). Smooth muscle-specific TMEM16A expression protects against angiotensin II-induced cerebrovascular remodeling via suppressing extracellular matrix deposition. J. Mol. Cell. Cardiol. 134, 131–143. 31301303 10.1016/j.yjmcc.2019.07.002

[mjaa080-B96] Zhang J.Z. , Chen D., Lv L.Q., et al (2018). miR-448-3p controls intracranial aneurysm by regulating KLF5 expression. Biochem. Biophys. Res. Commun. 505, 1211–1215. 30322616 10.1016/j.bbrc.2018.10.032

[mjaa080-B97] Zhang J. , Zheng B., Zhou P. P., et al (2014). Vascular calcification is coupled with phenotypic conversion of vascular smooth muscle cells through Klf5-mediated transactivation of the Runx2 promoter. Biosci. Rep. 34, e00148. 25205373 10.1042/BSR20140103 PMC4219426

[mjaa080-B98] Zhang M.L. , Zheng B., Tong F., et al (2017a). iNOS-derived peroxynitrite mediates high glucose-induced inflammatory gene expression in vascular smooth muscle cells through promoting KLF5 expression and nitration. Biochim. Biophys. Acta Mol. Basis Dis. 1863, 2821–2834. 28711598 10.1016/j.bbadis.2017.07.004

[mjaa080-B99] Zhang X.H. , Zheng B., Han M., et al (2009). Synthetic retinoid Am80 inhibits interaction of KLF5 with RARα through inducing KLF5 dephosphorylation mediated by the PI3K/Akt signaling in vascular smooth muscle cells. FEBS Lett. 583, 1231–1236. 19292987 10.1016/j.febslet.2009.03.016

[mjaa080-B100] Zhang X.H. , Zheng B., Yang Z., et al (2015). TMEM16A and myocardin form a positive feedback loop that is disrupted by KLF5 during Ang II-induced vascular remodeling. Hypertension 66, 412–421. 26077572 10.1161/HYPERTENSIONAHA.115.05280

[mjaa080-B101] Zhang X. , Liu F., Wang Q., et al (2017b). Overexpressed microRNA-506 and microRNA-124 alleviate H_2_O_2_-induced human cardiomyocyte dysfunction by targeting krüppel-like factor 4/5. Mol. Med. Rep. 16, 5363–5369. 28849090 10.3892/mmr.2017.7243 PMC5647069

[mjaa080-B102] Zhang Y.N. , Xie B.D., Sun L., et al (2016). Phenotypic switching of vascular smooth muscle cells in the ‘normal region’ of aorta from atherosclerosis patients is regulated by miR-145. J. Cell. Mol. Med. 20, 1049–1061. 26992033 10.1111/jcmm.12825 PMC4882986

[mjaa080-B103] Zhang Z. , Teng C.T. (2003). Phosphorylation of Krüppel-like factor 5 (KLF5/IKLF) at the CBP interaction region enhances its transactivation function. Nucleic Acids Res. 31, 2196–2208. 12682370 10.1093/nar/gkg310 PMC153738

[mjaa080-B104] Zhao D. , Zheng H.-Q., Zhou Z., et al (2010). The Fbw7 tumor suppressor targets KLF5 for ubiquitin-mediated degradation and suppresses breast cell proliferation. Cancer Res. 70, 4728–4738. 20484041 10.1158/0008-5472.CAN-10-0040

[mjaa080-B105] Zhao X. S. , Zheng B., Wen Y., et al (2019). Salvianolic acid B inhibits Ang II-induced VSMC proliferation in vitro and intimal hyperplasia in vivo by downregulating miR-146a expression. Phytomedicine 58, 152754. 31009837 10.1016/j.phymed.2018.11.014

[mjaa080-B106] Zheng B. , Han M., Shu Y. N., et al (2011). HDAC2 phosphorylation-dependent Klf5 deacetylation and RARα acetylation induced by RAR agonist switch the transcription regulatory programs of p21 in VSMCs. Cell Res*.* 21, 1487–1508. 21383775 10.1038/cr.2011.34 PMC3193446

[mjaa080-B107] Zheng B. , Yin W.N., Suzuki T., et al (2017). Exosome-mediated miR-155 transfer from smooth muscle cells to endothelial cells induces endothelial injury and promotes atherosclerosis. Mol. Ther. 25, 1279–1294. 28408180 10.1016/j.ymthe.2017.03.031 PMC5475247

[mjaa080-B108] Zheng B. , Zheng C.Y., Zhang Y., et al (2018). Regulatory crosstalk between KLF5, miR-29a and Fbw7/CDC4 cooperatively promotes atherosclerotic development. Biochim. Biophys. Acta Mol. Basis Dis. 1864, 374–386. 29074464 10.1016/j.bbadis.2017.10.021

[mjaa080-B109] Zheng H.Q. , Zhou Z., Huang J., et al (2009). Krüppel-like factor 5 promotes breast cell proliferation partially through upregulating the transcription of fibroblast growth factor binding protein 1. Oncogene 28, 3702–3713. 19668233 10.1038/onc.2009.235

[mjaa080-B110] Zhi X. , Zhao D., Zhou Z., et al (2012). YAP promotes breast cell proliferation and survival partially through stabilizing the KLF5 transcription factor. Am. J. Pathol*.* 180, 2452–2461. 22632819 10.1016/j.ajpath.2012.02.025

[mjaa080-B111] Zhou Y. , Li Y., Xu S., et al (2019). Eukaryotic elongation factor 2 kinase promotes angiogenesis in hepatocellular carcinoma via PI3K/Akt and STAT3. Int. J. Cancer *146*, 1383–1395. 31286509 10.1002/ijc.32560

[mjaa080-B112] Zhu Y. , Takayama T., Wang B., et al (2017). Restenosis inhibition and re-differentiation of TGFβ/Smad3-activated smooth muscle cells by resveratrol. Sci. Rep. 7, 41916. 28165488 10.1038/srep41916 PMC5292946

